# Prevalence of sensitization to cow‘s milk in EuroPrevall Lithuanian birth cohort

**DOI:** 10.1186/2045-7022-3-S3-O21

**Published:** 2013-07-25

**Authors:** I Butiene, R Dubakiene, O Rudzeviciene

**Affiliations:** 1 Faculty of Medicine, Vilnius University, Vilnius, Lithuania; 2 Children's Hospital, Vilnius University, Vilnius, Lithuania

## Background

Although cow’s milk allergy is considered as food allergy prototype, true prevalence of cow's milk allergy in Lithuania is not known. Aims of this research were to determine the prevalence of sensitization to cow’s milk in EuroPrevall Lithuanian birth cohort, to evaluate the relationship between children age and gender, sensitization to other allergens, relationship between sensitization to food allergens and severity of atopic dermatitis, to determine the influence of different parental factors on sensitization to milk.

## Methods

Data analysis of sensitized to cow’s milk and their control children from Lithuanian birth cohort was performed. Sensitized to cow’s milk were grouped into 4 groups according to age: from birth to 6 months – group 1, 6–12 months – group 2, 12–18 months – group 3, more than 18 months of age – group 4.

## Results

The sensitization to cow’s milk was found in 3.4% (52/1558) of children. The significant difference between symptomatic and control children according gender was not detected (p = 0.34), but the analysis according the gender and age groups showed statistically significant difference (p=0.02): in group 1 sensitization was found in 80% of boys and only 20% of girls, while in group 4 - in 20% of boys and in 80% of girls. Sensitization to more than one allergen was found in 50% of sensitized to cow‘s milk children. 45 (86.5%) sensitized children were diagnosed with atopic dermatitis. The mean SCORAD index (35.5) for those children, who were sensitized to more than one food allergen, was significantly higher than sensitized only to milk – mean SCORAD index was 19.3 (p=0.006). Maternal disease during pregnancy and delivery, use of antibiotics, parental allergic diseases had no significant influence on early sensitization to cow‘s milk (p>0.05). Maternal avoidance of milk products during pregnancy and lactation, as well as use in elevated amounts was not related to an early sensitization to milk allergens (p>0.05).

## Conclusion

The prevalence of sensitization to cow‘s milk in EuroPrevall Lithuanian birth cohort was 3.4%. In children less than 6 months the sensitization rate was significantly higher in boys, and for older than 18 months – in girls. In children, sensitized to more than one food allergen, SCORAD index was significally higher comparing to sensitized only to milk. There were no statistically significant relationships between maternal disease, use of antibiotic, maternal diet during pregnancy and lactation, parental allergy and sensitization to milk.

## Disclosure of interest

None declared.

